# RNF219 interacts with CCR4–NOT in regulating stem cell differentiation

**DOI:** 10.1093/jmcb/mjaa061

**Published:** 2020-10-26

**Authors:** Hao Du, Chen Chen, Yan Wang, Yang Yang, Zhuanzhuan Che, Xiaoxu Liu, Siyan Meng, Chenghao Guo, Manman Xu, Haitong Fang, Fengchao Wang, Chengqi Lin, Zhuojuan Luo

**Affiliations:** 1 School of Life Science and Technology, The Key Laboratory of Developmental Genes and Human Disease, Southeast University, Nanjing 210096, China; 2 Department of Biological Sciences, Center for Systems Biology, the University of Texas at Dallas, Richardson, TX 75080, USA; 3 Institute of Combined Injury of PLA, State Key Laboratory of Trauma, Burns and Combined Injury, Army Medical University, Chongqing 400038, China; 4 Co-innovation Center of Neuroregeneration, Nantong University, Nantong 226001, China

**Keywords:** RNF219, CCR4–NOT, protein complex, stem cell differentiation, deadenylation

## Abstract

Regulation of RNA stability plays a crucial role in gene expression control. Deadenylation is the initial rate-limiting step for the majority of RNA decay events. Here, we show that RING finger protein 219 (RNF219) interacts with the CCR4–NOT deadenylase complex. RNF219–CCR4–NOT exhibits deadenylation activity *in vitro*. RNA-seq analyses identify some of the 2-cell-specific genes and the neuronal genes significantly downregulated upon RNF219 knockdown, while upregulated after depletion of the CCR4–NOT subunit CNOT10 in mouse embryonic stem (ES) cells. RNF219 depletion leads to impaired neuronal lineage commitment during ES cell differentiation. Our study suggests that RNF219 is a novel interacting partner of CCR4–NOT and required for maintenance of ES cell pluripotency.

## Introduction

Regulation of RNA metabolism is essential in various biological processes. RNA decay is the last but critical step to control RNA in both quantity and quality. Nearly all eukaryotic messenger RNAs (mRNAs) and long non-coding RNAs (lncRNAs) are protected by 7-methylguanosine (m^7^G) cap at the 5′-end and poly-adenine (poly(A)) tail at the 3′-end from degradation by exonucleases. RNA decay is usually triggered by transcript deprotection, such as decapping or deadenylation. In eukaryotes, deadenylation, or poly(A) tail shortening and removal, is the preferred initiating event for the vast majority of mRNAs and lncRNAs to be fated to rapid decay. After the rate-limiting deadenylation, unprotected bodies of RNA can be attacked at both ends by the 5′–3′ or 3′–5′ degradation machineries. In addition to mRNA and lncRNA decay, deadenylation is also vital for translational silencing.

To date, three types of deadenylase have been identified in mammals, including the CCR4–NOT complex, the PAN2–PAN3 complex, and PARN (Goldstrohm and Wickens, 2008). PARN maintains the short (A) tails of mRNAs in oocytes to control translation in a dormant state (Kim and Richter, 2006) and also functions in the maturation of small nucleolar RNA (Berndt et al., 2012). PAN2–PAN3 is inefficient in trimming the final 20–25 adenosines of the poly(A) tail, thus minimally degrading the body of transcript (Lowell et al., 1992; Wolf et al., 2014). However, PAN2–PAN3 is able to function together with CCR4–NOT, which is the major deadenylase, to remove the poly(A) tail thoroughly and stimulate RNA decay (Yamashita et al., 2005). CCR4–NOT is a highly conserved multi-subunit complex, which contains two deadenylases, CNOT7 (or its paralogue CNOT8) and CNOT6 (or its paralogue CNOT6L) (Lau et al., 2009). These two catalytic components differ in substrates: CNOT7 prunes poly(A) tail free of poly(A)-binding protein (PABP), while CNOT6 dislodges protective PABP from the A tails, subsequently leading to deadenylation (Webster et al., 2018; Yi et al., 2018). CNOT1, the largest component of CCR4–NOT, interacts with most of the other components and serves as a scaffold required for the complex assembly. The RING finger containing CNOT4 possesses ubiquitin ligase activity in both yeast and human. CNOT4 controls the proteasome-dependent degradation of multiple chromatin-related proteins, such as the transcription regulator PAF1 and H3K4me3 demethylase JARID1C (Mersman et al., 2009; Sun et al., 2015). Although the precise roles played by CNOT4 and other non-enzymatic components within CCR4–NOT remain to be elucidated, a growing body of evidence manifests that these components are indispensable for regulation of gene expression (Mulder et al., 2007; Kruk et al., 2011; Venters et al., 2011).

CCR4–NOT can be recruited to different subclasses of RNAs via different mechanisms. CCR4–NOT was found to engage in the deadenylation of RNAs with microRNA (miRNA) target sites by the miRNA-induced silencing complex (miRISC) complex (Braun et al., 2011; Fabian et al., 2011; Chekulaeva et al., 2011). N6-methyladenosine (m^6^A) reader protein YTHDF2 recruits CCR4–NOT to deadenylate RNAs with m^6^A modifications (Du et al., 2016). CCR4–NOT interacts with Tristetraprolin (TTP), which is an AU-rich element (ARE) containing RNA-binding protein, to deadenylate RNAs with AREs (Lykke-Andersen and Wagner, 2005; Fabian et al., 2013). The SMG5–SMG7 heterodimer recruits CCR4–NOT, thus triggering non-sense-mediated mRNA decay to rapidly degrade aberrant mRNAs bearing premature translation termination codons (Loh et al., 2013). However, other than these recruiting partners, factors involved in deadenylation through interacting with CCR4–NOT remain largely unknown.

RING finger protein 219 (RNF219), containing an evolutionarily conserved RING finger domain at its N-terminus, is a poorly characterized ubiquitin ligase. A recent study shows that RNF219 is able to promote DNA replication origin firing through ubiquitination of the origin recognition complex (Coulombe et al., 2019). Here, we biochemically identified RNF219 as an interacting factor of CCR4–NOT. RNA levels of some of the 2-cell-specific genes and the neuronal genes undergo the opposite regulations by RNF219 and the CCR4–NOT subunit CNOT10 in mouse embryonic stem (ES) cells. While the main subunits of CCR4–NOT are essential in preserving ES cell identity (Zheng et al., 2012; Zukeran et al., 2016), we found here that depletion of RNF219 in ES cells leads to impaired neuronal lineage commitment. Our study suggests RNF219 as a cofactor of CCR4–NOT to regulate transcript levels and also provides a novel direction in studying the pathological processes of RNF219-linked human diseases.

## Results

### Interaction of RNF219 with the CCR4–NOT complex

**Figure 1:**
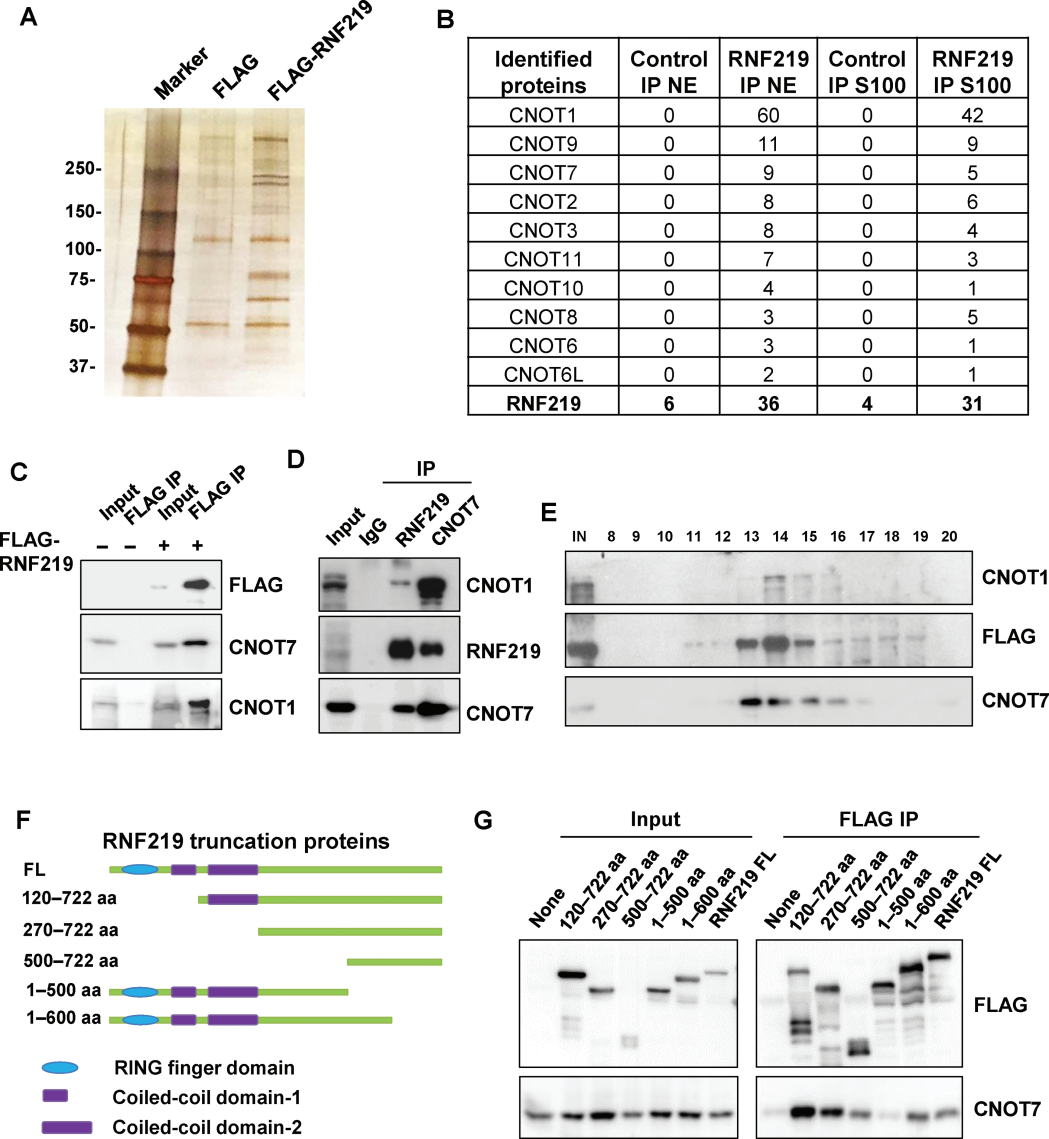
RNF219 interacts with the CCR4–NOT complex. (**A**) FLAG-purification of RNF219. Stable cell line expressing FLAG-tagged RNF219 was generated in HEK-293 FIT cells. The FLAG-RNF219-assoicated proteins were purified from the FLAG-RNF219-expressing cells using the FLAG-affinity purification method and analyzed by SDS–PAGE and silver staining. (**B**) Mass spectrometry identified the components of CCR4–NOT in the FLAG-RNF219 purification. S100, cytoplasmic extract; NE, nuclear extract. (**C**) Validation of the interaction of RNF219 with CNOT1 and CNOT7 by FLAG immunoprecipitation. (**D**) Endogenous co-IP of RNF219 and CNOT7 from HEK-293 cells. (**E**) Size exclusion chromatography of cytoplasmic extracts from the FLAG-RNF219 stable cell line demonstrating that majority of RNF219 co-eluted with the components of CCR4–NOT at ∼1.9 MDa (fractions 13–15). (**F**) Schematic diagram of RNF219-truncated proteins used in **G**. (**G**) FLAG immunoprecipitations mapping the interaction domain of RNF219 with CNOT7. HEK-293 cells transfected with a plasmid encoding each of the FLAG-tagged RNF219-truncated protein (**F**) were subjected to cell lysis and FLAG immunoprecipitation.

In order to investigate the function of RNF219, we sought to identify factors that interact with RNF219. First, a tetracycline inducible stable cell line expressing N-terminal FLAG-tagged RNF219 was generated by using the HEK-293 Flp-In-TRex (FIT) system. FLAG affinity purification was performed in the Benzonase nuclease-treated condition to avoid nucleic acids-dependent protein–protein interactions. FLAG-RNF219 and control purifications were then resolved by SDS/PAGE prior to silver staining (Figure 1A). Analyses of independent purifications from both nuclear and cytoplasmic S100 extracts led to the identification of nearly all the subunits of CCR4–NOT, except CNOT4 (Figure 1B). In addition to functioning in translation inhibition and deadenylation in cytoplasm, CCR4–NOT is also involved in RNA synthesis in nucleus at different stages, such as modification of the chromatin template and regulation of RNA polymerase II processivity (Mulder et al., 2007; Sun et al., 2015). Thus, it is expected that the interaction between RNF219 and CCR4–NOT can be detected in both cytoplasm and nucleus.

The co-purification of RNF219 with the core components of CCR4–NOT, CNOT1 and CNOT7, were validated by western blotting (Figure 1C). Co-immunoprecipitation (co-IP) using the antibody specific to RNF219 or CNOT7 further confirmed the endogenous interaction between RNF219 and the CCR4–NOT components (Figure 1D;  Supplementary Figure S1A and B). In addition, co-fractionation of RNF219 with the CCR4–NOT components was examined by applying cytoplasmic and nuclear extracts from the HEK-293 FIT cells stably expressing FLAG-RNF219 to size exclusion chromatography. Western blotting analyses revealed that the majority of RNF219 co-eluted with CNOT1 and CNOT7 at ∼1.9 MDa (fraction 13–15; Figure 1E;  Supplementary Figure S1C). Together, these results suggested that RNF219 is able to associate with CCR4–NOT. Our finding is consistent with a recent study, which independently revealed the interaction between RNF219 and CCR4–NOT in HeLa cells (Guénolé et al., 2019).

The RNF219 protein consists of an N-terminal RING domain, two middle coiled-coil domains, and a C-terminal uncharacterized region (Figure 1F). A series of FLAG-tagged RNF219-truncated proteins were used to map the CCR4–NOT interaction domain in RNF219. No loss of binding to CNOT7 was observed when truncating both the RING and coil-coil domains (Figure 1G). The 1–600 amino acids (aa) C-terminal deletion mutant of RNF219 was able to interact with CNOT7, while the 1–500 aa mutant failed to co-immunoprecipitate CNOT7 (Figure 1G). Deletion of 500–600 aa in RNF219 greatly impaired RNF219 interaction with CCR4–NOT (Supplementary Figure S1D), suggesting that the 500–600 aa of RNF219 located in the center of the C-terminal region might mediate the interaction with CCR4–NOT.

### Deadenylation activity of RNF219–CCR4–NOT

**Figure 2:**
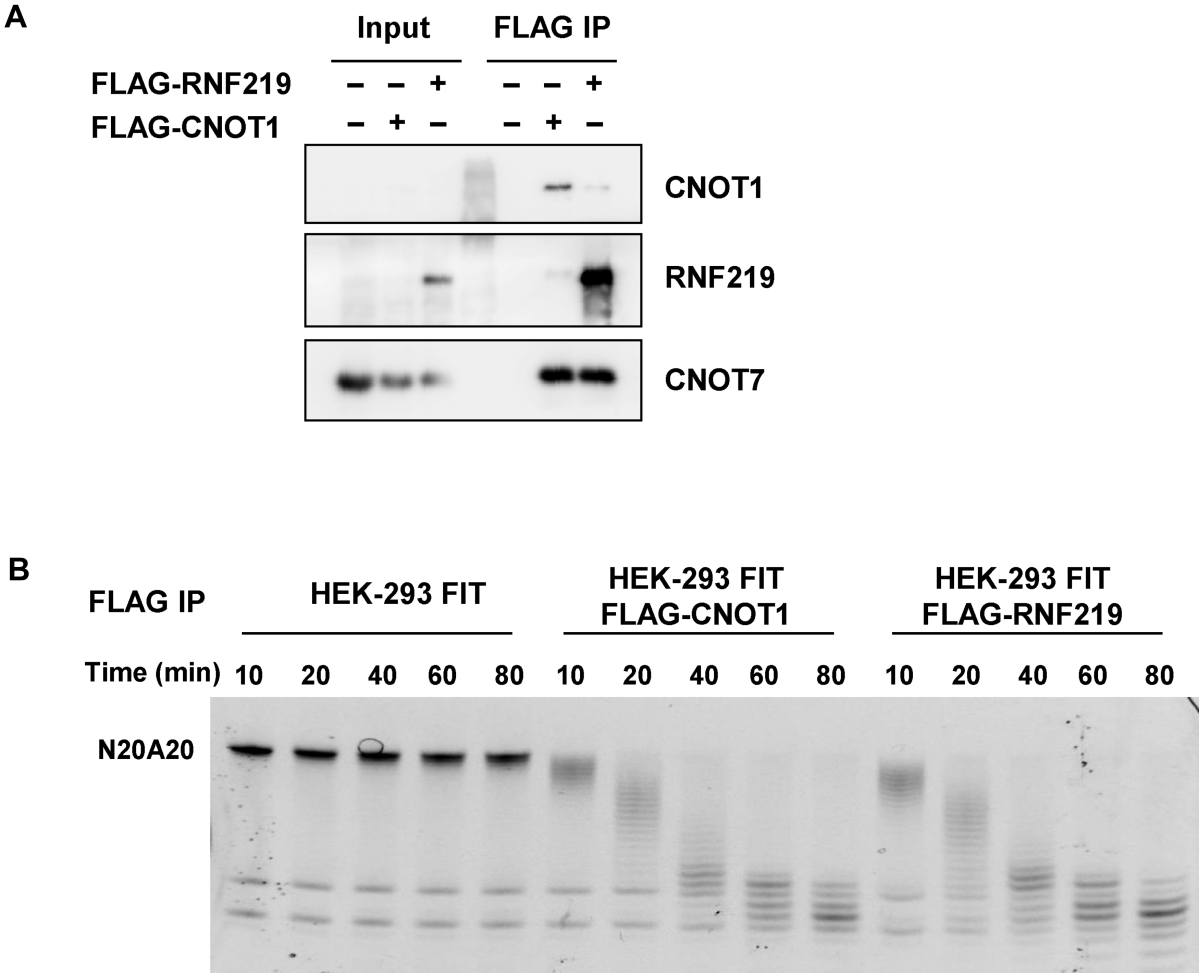
RNF219–CCR4–NOT exhibits deadenylation activity. (**A**) FLAG purification of RNF219 from the HEK-293 FIT cells stably expressing FLAG-tagged RNF219 and CNOT1 from the FLAG-CNOT1-expressing HEK-293 FIT cells. Levels of RNF219, CNOT1, and CNOT7 in the purified immuneprecipitates were examined by western blotting. (**B**) *In vitro* deadenylation of the synthesized N20A20 RNA using the affinity-purified FLAG-RNF219 or FLAG-CNOT1-containing immunoprecipitate. Samples were harvested at the indicated time intervals and resolved by electrophoresis separation. FLAG purification from HEK-293 FIT cell lysate was used as a negative control.

Given that CCR4–NOT is the major deadenylase in eukaryotic cells, we further explored whether RNF219 is also involved in deadenylation and RNA stability regulation. In order to examine whether RNF219-bound CCR4–NOT possesses deadenylation activity, we performed FLAG-CNOT1 and FLAG-RNF219 immuno-affinity purification from HEK-293 FIT cell lysates, respectively. The purified immune-precipitates were analyzed by western blotting, and the catalytic subunit of CCR4–NOT, CNOT7, was co-purified with both FLAG-CNOT1 and FLAG-RNF219 (Figure 2A). *In vitro* deadenylation assay indicated that the affinity-purified FLAG-RNF219-containing immunoprecipitate exhibited deadenylation activity toward the synthesized N20A20 RNA (Figure 2B).

### Regulation of deadenylation by RNF219

**Figure 3:**
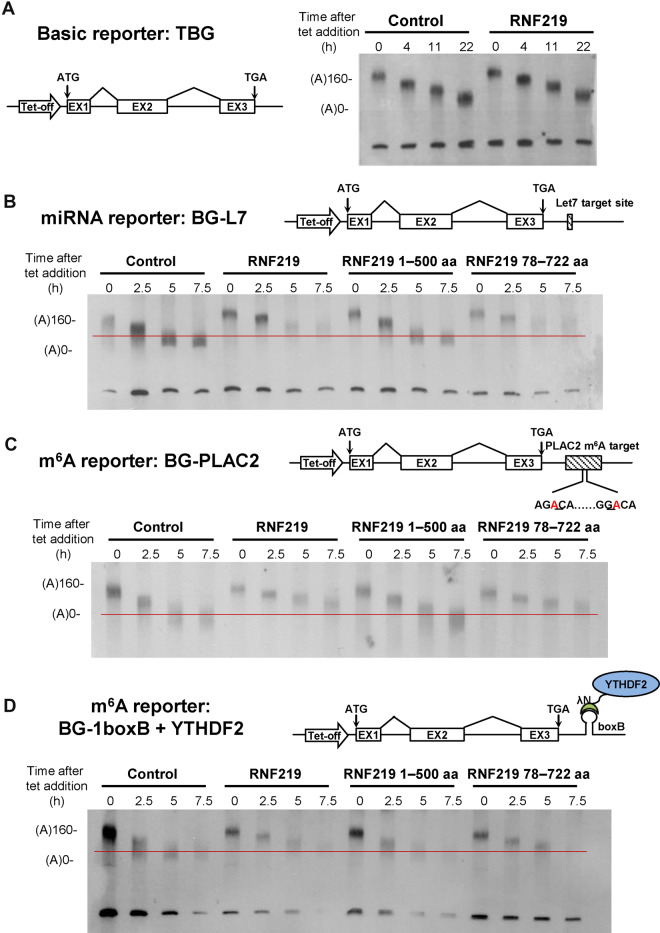
RNF219 affects deadenylation mediated by miRNA and m^6^A modification. (**A**) Schematic diagram of the basic reporter TBG construct. Blank boxes indicate exons (left panel). Deadenylation assay of TBG in control and RNF219-overexpressing cells (right panel). Tetracycline was briefly removed from the culture medium, thus leading to the production of a homogenous amount of BG mRNAs. Cells were harvested at the indicated time intervals for cytoplasmic RNA extraction. RNA samples were subjected to RNaseH cleavage, followed by electrophoresis separation and northern blotting analyses. (**B**–**D**) Schematic diagrams of the miRNA reporter (**B**), the m^6^A reporter (**C**), and the YTHDF2 tethered reporter (**D**) constructs (upper panels). Deadenylation assay of each reporter following the overexpression of RNF219 full-length, 1–500 aa, and 78–722 aa (lower panels).

To explore the role of RNF219 in a cellular context, we employed the transiently inducible b-globin (BG) reporter system combined with the transcriptional pulse-chase assay to analyze the effect of RNF219 on the deadenylation of RNA with different types of destabilization elements. As visualized by northern blotting, RNF219 overexpression significantly decelerated the deadenylation rate of the BG reporter that harbors the miRNA Let-7 target site (BG-L7) in the 3′ UTR (Figure 3B;  Supplementary Figure S2A). As a control, RNF219 overexpression seemed not obviously affect the deadenylation of 3′ fragments of the basic TBG reporter, which does not harbor any *cis*-element to promote deadenylation (Figure 3A). It has been previously established that the RISC complex can recruit CCR4–NOT to RNAs containing miRNA target sites. Here, we found that RNF219 was also able to interact with AGO2 in miRISC (Supplementary Figure S2B). Therefore, it might be possible that RNF219 could modulate miRNA-mediated deadenylation through physical interaction with CCR4–NOT and AGO2.

We next proceeded with the BG-PLAC2 reporter, whose 3′ UTR contains a fragment with two m^6^A consensus motifs from the lncRNA PLAC2 (Figure 3C). Similar to its role in BG-L7, RNF219 also inhibited the deadenylation of the m^6^A reporter BG-PLAC2. YTHDF2, the m^6^A-binding protein, is able to destabilize the m^6^A modified RNAs (Wang et al., 2014; Du et al., 2016). To our expectation, RNF219 overexpression impeded the deadenylation of the YTHDF2-tethered BG reporter (Figure 3D). Thus, RNF219 might also regulate m^6^A-mediated deadenylation.

To determine whether the role of RNF219 in the inhibition of deadenylation depends on its interaction with CCR4–NOT, we also carried out the deadenylation assay using the C-terminal deletion mutant of RNF219 (RNF219 1–500 aa), which lost the interaction with CCR4–NOT. As expected, RNF219 1–500 aa was incapable of slowing down the deadenylation rate of the transcripts of BG-L7, BG-PLAC2, and YTHDF2-tethered reporters (Figure 3B–D). Deletion of the CCR4–NOT interaction domain (500–600 aa) in RNF219 also diminished the inhibitory role of RNF219 on the deadenylation of the BG-PLAC2 transcripts (Supplementary Figure S2C). In contrast, the RING domain deletion mutant of RNF219 (RNF219 78–722 aa), which retained the interaction with CCR4–NOT, was able to decelerate the deadenylation rate of these reporter transcripts to a similar extent as wild-type RNF219 (Figure 3B–D). Thus, the inhibitory effect of RNF219 overexpression on deadenylation might require its interaction with CCR4–NOT.

### Opposite regulations of the 2-cell-specific genes by RNF219 and CNOT10 in mouse ES cells

**Figure 4:**
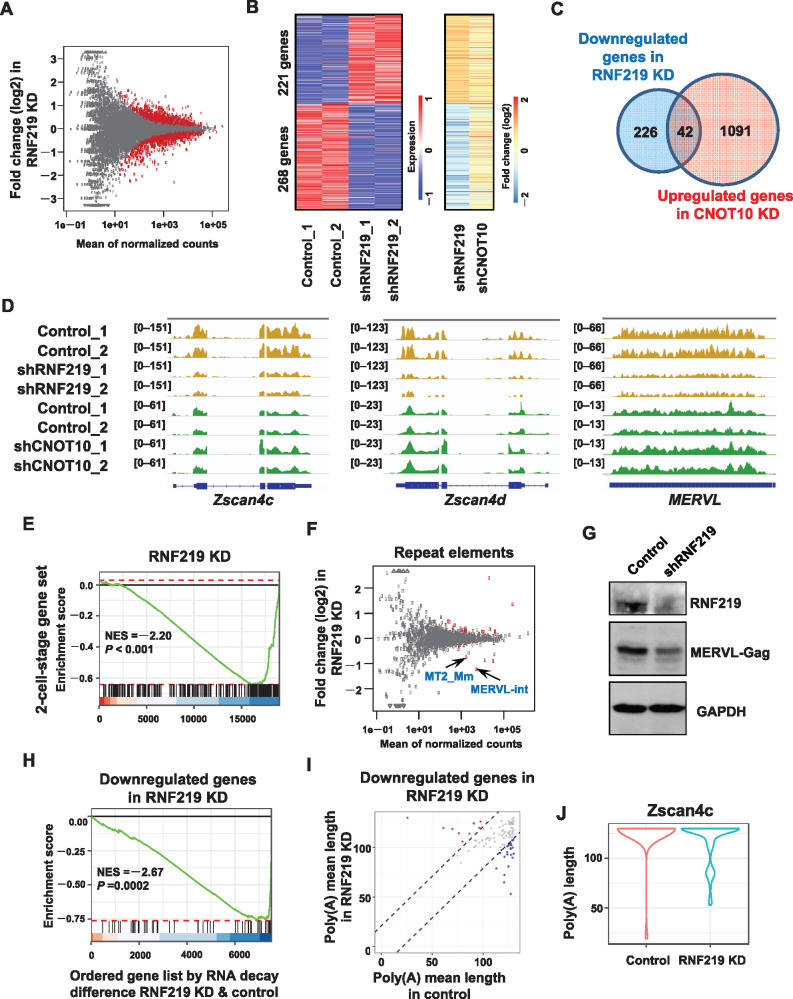
The 2-cell-specific genes are oppositely regulated by RNF219 and CNOT10 in mouse ES cells. (**A**) MA plot showing differential expression of genes after KD of RNF219 in mouse ES cells. The plot depicts the mean of normalized counts (x-axis) and log2 fold changes that calculated using DESeq2. The red dots represent genes with significance with the adjusted *P*-value <0.05. (**B**) Heat maps showing expression levels (left panel) and fold change (right panel) of differentially expressed genes (|Log2FC| > 0.58) in RNF219 KD mouse ES cells. The right panel also showing fold change of these genes in CNOT10 KD mouse ES cells. (**C**) The Venn diagram depicting the overlap of the downregulated genes after RNF219 KD and the upregulated genes after CNOT10 KD. |Log2FC| > 0.58. (**D**) Genome browse track file showing that the *Zscan4* family and *MERVL* are downregulated after RNF219 KD but upregulated after CNOT10 KD. (**E**) GSEA plot showing that the 2-cell-specific genes are highly enriched in the downregulated genes after RNF219 KD in mouse ES cells. (**F**) MA plot showing differential expression of repeat elements after KD of RNF219 in mouse ES cells. The plot depicts the mean of normalized counts (x-axis) and log2 fold changes that calculated using DESeq2. The red dots represent repeat elements with significance with the adjusted *P*-value <0.05. (**G**) Western blot showing the protein level of MERVL-Gag reduced after RNF219 KD in mouse ES cells. (**H**) GSEA plot showing that transcripts whose levels are maintained by RNF219 tend to be destabilized after RNF219 KD. (**I**) The scatter plot representing the geometric mean poly(A) tail length in control and RNF219 KD cells, as measured by mTAIL-seq, showing only the genes downregulated after RNF219 KD. (**J**) The violin plots showing the distribution of poly(A) tag length of *Zscan4c* in control and RNF219 KD cells.

In order to further explore the functional link between RNF219 and the CCR4–NOT complex, we performed differential RNA level analyses using RNA-seq upon shRNA-mediated knockdown (KD) of RNF219 or CNOT10 in mouse ES cells. Recently, reconstitution of recombinant CCR4–NOT revealed that the CNOT10: CNOT11 module is able to bind to RNA directly and stimulate deadenylation (Raisch et al., 2019). CNOT10 KD led to the RNA levels of 1133 genes upregulated and 353 genes downregulated (Supplementary Figure S3A and B), consistent with the role of CCR4–NOT in negative regulation of RNA level by deadenylation. However, in total, only 489 genes were significantly differentially expressed after RNF219 KD, with 268 genes downregulated (Figure 4A and B). These genes downregulated after RNF219 KD tended to be changed in the opposite direction after CNOT10 KD (Figure 4B). The RNA levels of 42 genes were significantly downregulated upon RNF219 KD whereas upregulated upon CNOT10 KD with fold change >1.5 (Figure 4C). For example, the *Zscan4* family genes, which are the specific markers for 2-cell-stage embryos and 2-cell state-like ES cells, were listed among the 42 RNF219–CNOT10 targets (Figure 4D;  Supplementary Figure S3C; Zalzman et al., 2010). Thus, RNF219 and CNOT10 might play opposite roles in controlling transcript level.

Interestingly, besides *Zscan4c* and *Zscan4d*, 44 of the rest of the genes downregulated after RNF219 KD were 2-cell-specific as well. Gene set enrichment analysis (GSEA) confirmed that the 2-cell-specific genes were preferentially downregulated upon RNF219 KD (Figure 4E). The *Zscan4* genes are located adjacent to the endogenous retrovirus *MERVL*. Reminiscent of the expression pattern of *Zscan4*, *MERVL* is also exclusively expressed in 2-cell embryos and 2-cell-like ES cells (Macfarlan et al., 2012). The regulation of 2-cell-specific gene transcript levels by RNF219 prompted us to further investigate whether *MERVL* transcripts are also targeted by RNF219. We thus analyzed the expression levels of the repeat elements, including *MERVL*, following the RNF219 KD by mapping RNA-seq reads to the consensus of different repeat elements. MA-plot analysis indicated that the expression levels of *MERVL* and *MERVL*-derived *LTR MT2 Mm* were also significantly downregulated following RNF219 KD (Figure 4D and F). Consistently, the expression level of MERVL-Gag protein was also reduced in RNF219 KD ES cells (Figure 4G).

Give that CCR4–NOT is the major deadenylase in eukaryotic cells, we further explored whether RNF219 regulates the RNA levels through affecting deadenylation and subsequent decay. We first estimated differential RNA decay rate from RNA-seq data in RNF219 KD mouse ES cells. Transcripts whose levels maintained by RNF219 were highly enriched in the list of transcripts, which were destabilized upon RNF219 KD (Figure 4H). We next performed mTAIL-seq to measure whether the poly(A) tail length was altered upon RNF219 KD. mTAIL-seq analysis indicated that these RNF219 target transcripts tended to have shorter mean poly(A) tail length after RNF219 KD (Figure 4I). For example, poly(A) tag distribution demonstrated that the shorter poly(A) tags of *Zscan4c* were significantly increased after RNF219 KD (Figure 4J).

### Requirement of RNF219 for neuronal specification of mouse ES cells

**Figure 5:**
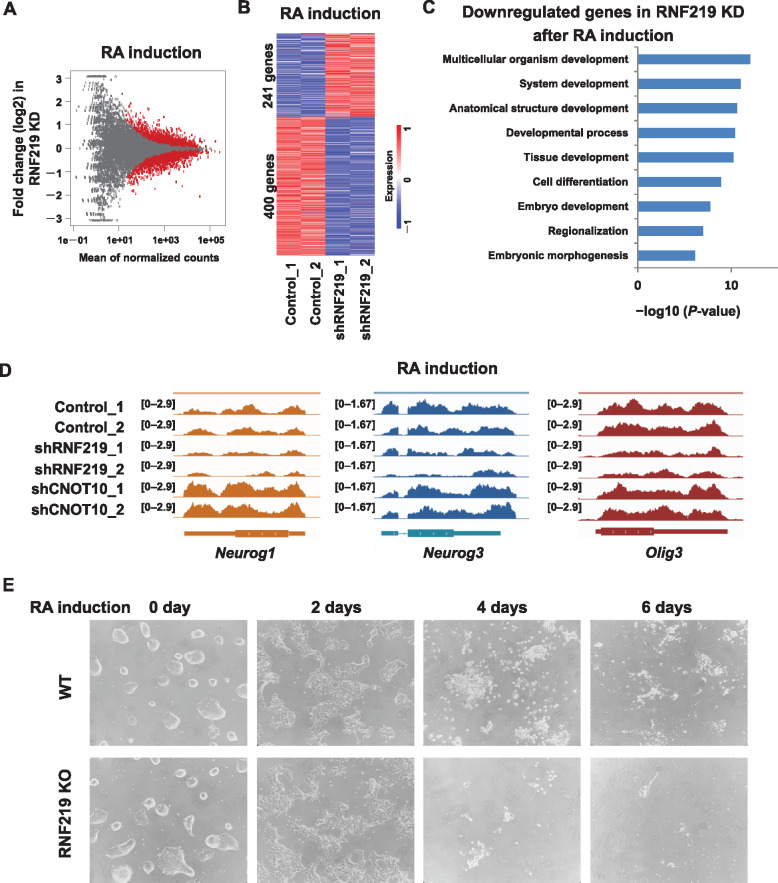
RNF219 is essential for neuronal specification of mouse ES cells. (**A**) MA plot showing differential expression of genes after RNF219 KD in mouse ES cells treated with RA. The plot depicts the mean of normalized counts (x-axis) and log2 fold changes that calculated using DESeq2. The red dots represent genes with significance with the adjusted *P*-value <0.05. (**B**) Heat maps showing expression levels of differentially expressed genes after RNF219 KD in mouse ES cells treated with RA. |Log2FC| > 0.58. (**C**) Functional annotation using DAVID of the downregulated genes after RNF219 KD in mouse ES cells treated with RA. (**D**) Genome browser track files showing that *Neurog1*, *Neurog3*, and *Olig3* are downregulated after RNF219 KD but upregulated after CNOT10 KD in mouse ES cells treated with RA. (**E**) Monolayer differentiation of wild-type (WT) and RNF219 KO mouse ES cells in N2B27 medium for indicated days.

To investigate whether RNF219 is involved in controlling transcript levels during ES cell differentiation, we performed differential RNA level analyses in retinoid acid (RA)-exposed mouse ES cells for neuronal differentiation after RNF219 KD. RNA-seq analyses identified 400 genes downregulated and 241 genes upregulated in RA-treated mouse ES cells after RNF219 KD (Figure 5A and B). The levels of the transcripts from the *Zscan4* family and *MERVL* were also maintained by RNF219 in differentiated ES cells (Supplementary Figure S4A). Further functional annotation of using DAVID indicated that genes involved in developmental processes and cell differentiation were highly enriched in the downregulated gene list (Figure 5C). For example, the RNA levels of the known neuronal genes *Neurog1*, *Neurog3*, and *Olig3* were reduced after RNF219 KD in RA-treated mouse ES cells (Figure 5D). Interestingly, CNOT10 KD upregulated the RNA levels of these genes in RA-differentiated cells. To examine the requirement of RNF219 in neuronal specification, an RNF219 knockout (KO) ES cell line was generated and assessed for differentiation ability in N2B27 media (Supplementary Figure S4B; Ying et al., 2003). The RNF219 KO ES cells displayed typical ES morphology under 2i/LIF culture condition (Figure 5E). However, the cells cannot be maintained and differentiated after 4 days of culture in the N2B27 differentiation media. Our results indicated that RNF219 could be an essential factor for neuronal differentiation of ES cells.

## Discussion

CCR4–NOT is the major deadenylase in mammals. We here demonstrated that RNF219 interacts with CCR4–NOT and functions in ES cell pluripotency maintenance. RNF219 is required for maintaining the proper expression levels of some of the 2-cell-specific genes and the neuronal genes in mouse ES cells. RNF219 KD impairs the capability of ES cells in neuronal differentiation. Our study thus could highlight a potential direction in investigating the causes of RNF219-related diseases in nervous system.

The interplay between miRISC and CCR4–NOT has been intensively studied (Fabian and Sonenberg, 2012; Jonas and Izaurralde, 2015). AGO and GW182 are the two core components of miRISC. Following facilitation of miRNA pair with 3′ UTR of target RNA by AGO, GW182, or its human paralog TNRC6, recruits CCR4–NOT through interacting with the scaffold CNOT1 (Braun et al., 2011; Chekulaeva et al., 2011). In this study, we found that RNF219 can interact with both CCR4–NOT and AGO2, suggesting that a potential double locker mechanism to consolidate miRISC with CCR4–NOT in controlling miRNA-mediated deadenylation. Intriguingly, CNOT4, the core subunit of the canonical CCR4–NOT, was not detected in our FLAG-RNF219 purification by mass spectrometry. A recent study also reported that CNOT4 was not identified in the purified RNF219-containing complexes (Guénolé et al., 2019). As both CNOT4 and RNF219 are RING domain-containing factors possessing E3 ligase activity, we postulated that CNOT4 and RNF219 might be mutually exclusive and that different versions of CCR4–NOT could exist with functional diversity and target specificity.

Opposite regulations by RNF219 and CNOT10 in transcript levels suggest that the deadenylation machinery might be multi-layered with buffering element, which might be of crucial importance to development. For example, *Zscan4* and *MERVL*, whose transcripts are identified in the current study as the *in vivo* targets of RNF219 and CNOT10, are essential for maintenance of telomeres, genome stability, and even totipotent state of 2-cell state-like ES cells (Zalzman et al., 2010; Macfarlan et al., 2012). Although only sporadic ES cells in culture are ZSCAN4 and MERVL-positive at a given time, nearly every ES cell oscillates between ZSCAN4^+^MERVL^+^ and ZSCAN4^−^MERVL^−^ states after prolonged passaging (Zalzman et al., 2010; Macfarlan et al., 2012), suggesting that dramatically rapid turnover of the *MERVL* and *Zscan4* transcripts occurs. Slowing down the deadenylation rate of these transcripts by RNF219 might be prone to prevent precocious decay, thus creating a time window enough for MERVL and ZSCAN4 to execute their functions during ES cell maintenance and early embryo development.

## Materials and methods

### Antibodies

Antibody against RNF219 was generated in our laboratory. Antibody against CNOT1 was purchased from Proteintech (14276-1-AP). Antibody against CNOT7 was purchased from Abcam (ab195587). Antibody against MERVL-Gag was purchased from Beyotime (AF0240). Antibody against AGO2 was purchased from Millipore (MABE253). Antibodies recognizing TUBULIN, GAPDH, FLAG, V5, and HA, respectively, were obtained from Sigma.

### Plasmids and cell culture

pV5-CNOT7, pV5-CNOT6, pHA-CNOT1, TBG, BG-L7, BG-PLAC2, BG-1boxB, and pNF-YTHDF2 were previously described (Du et al., 2016). FLAG-tagged RNF219 and CNOT1 cDNAs were cloned into pCDNA5/FRT-TO vector (Invitrogen). The expression vectors were then transfected into HEK-293 FIT cells and followed by selection with hygromycin to generate stable cell lines. HeLa-tTA cells were purchased from Clontech. V6.5 mouse ES cells were cultured in serum and leukemia inhibitory factor (LIF)-supplemented medium on irradiated mouse embryonic fibroblasts. All cells were maintained at 37°C under 5% CO_2_.

Lentivirus-mediated RNAi was previously described (Lin et al., 2011). Seventy-two hours after lentiviral infection, ES cells were treated with or without RA for 24 h before harvesting. For all analyses, cells were grown for one passage off feeders on tissue culture plates for 30 min. RNF219 KO V6.5 ES cell line was generated by CRISPR–Cas9 genome editing technique. For neuronal differentiation, mouse ES cells were cultured in N2B27 medium and treated with RA for 6 days.

### Flag purification, immunoprecipitation, and size exclusion chromatography

Flag purification was performed as previously described (Lin et al., 2010; Luo et al., 2012). Briefly, the expression of FLAG-RNF219 was induced with doxycycline for 48 h. The protein complexes were purified using the FLAG-affinity purification approach in the presence of benzonase (Sigma) and analyzed by SDS–PAGE and silver staining before subjected to mass spectrometry.

Immunoprecipitation was performed as previously described (Lin et al., 2010; Luo et al., 2012). Briefly, cells were lysed in 420 mM NaCl containing lysis buffer supplemented with protease inhibitor cocktail (Sigma) at 4°C. After centrifugation, the balance buffer (20 mM HEPES, pH 7.4, 1 mM MgCl_2_, and 10 mM KCl) was added to the supernatant to make the final NaCl concentration at 300 mM. The supernatant was then incubated with antibodies and protein A beads overnight at 4°C. The beads were spun down and washed three times with the wash buffer before boiling in the SDS loading buffer.

Size exclusion chromatography was performed as previously described (Lin et al., 2010; Luo et al., 2012). Nuclear and cytoplasmic extracts were subjected to Superose 6 size exclusion chromatography (GE Healthcare) with size exclusion buffer (40 mM HEPES, PH 7.5, 350 mM NaCl, 10% glycerol, and 0.1% Tween-20). Fractions were resolved in SDS–PAGE gels, followed by western blotting.

### In vitro deadenylation assay

HEK-293 FIT cells stably expressing FLAG-CNOT1 or FLAG-RNF219 were subjected to FLAG purification. The FLAG-M2 beads-bound bait protein and its interacting factors were incubated with 200 nM FAM-N20A20 synthetic RNA substrate in 50 mM Tris-HCl (pH 7.5), 50 mM NaCl, 2 mM MgCl_2_, and 0.5 mM DTT in a rotator at 37°C for 30 min. Samples were collected at different time points and analyzed on a 20% polyacrylamide denaturing gel with 7 M urea. The images were analyzed with a fluorescence imager.

### Reporter deadenylation assay

Deadenylation assay of BG reporters was previously described (Du et al., 2016) with pcDNA5-RNF219 or truncations overexpressed. HeLa-tTA cells were plated on a 6-well plate one day before transfection in DMEM containing 20 ng/ml tetracycline. One microgram of the reporter plasmid and 500 ng of RNF219 or control plasmid were used for transfection. The transcription of BG mRNA was induced by removing tetracycline 12 h after transfection, and 3 h after induction, tetracycline was added to a final concentration of 1 μg/ml to block the transcription of BG. Cytoplasmic RNAs were then isolated at various time intervals. RNAs were treated with RNaseH in the presence of an antisense DNA oligo (5′-GTCCAGGTGACTCAGACCCTC-3′ for TBG, BG-L7, and BG-1boxB; 5′-CCAGCCACCACCTTCTGATAGGC-3′ for BG-PLAC2). The digested RNA samples were analyzed by electrophoresis (5.5% PAGE with 8 M urea) and northern blotting using the DIG Northern Starter Kit (Roche). 

### RNA-seq data processing

We first evaluated each RNA-seq dataset quality using FastQC (version 0.11.8) and confirmed that all datasets are qualified for following analysis. The RNA-seq reads were aligned to mouse reference genome (mm10/GRCm38) using HISAT2 (version 2.1.0). The mouse reference genome sequence was downloaded from ENSEMBL (Mus Musculus GRCm38/mm10). Then, we used featureCounts (version 2.0.0) to count reads or read pairs for each protein-coding gene annotated in GENCODE (vM23) (Liao et al., 2014). Besides, we also counted reads or read pairs for repeat elements annotated in RepeatMasker. Then, we conducted differential expression analysis using DESeq2 (Love et al., 2014) and selected the |log2FoldChange| > 1.5 and FDR < 0.05 as the cutoff to identify significantly differentially expressed genes.

### Identification of 2-cell-stage genes

To identify those genes that were specifically expressed in mouse 2-cell stage, we downloaded a single-cell RNA-seq dataset (GSE53386) from GEO (Fan et al., 2015). The gene expression profiles (FPKM) of oocyte, zygote, 2-cell, 4-cell, and 8-cell. Blastocyst and morula were collected. Then, we calculated a specificity ratio for each gene and selected specificity ratio >1 as the cutoff to identity cell type-specific genes for each cell stage.

### RNA stability analysis

We used REMBRANDTS to estimate differential mRNA stability using RNA-seq data across multiple samples. Δexon–Δintron was used as an estimate of differential mRNA stability in several recent studies (Gaidatzis et al., 2015; Gosline et al., 2016). REMBRANDTS is designed to estimate gene-specific bias function that is then subtracted from Δexon–Δintron to provide unbiased differential mRNA stability measure (Alkallas et al., 2017). The coordinates of exonic and intronic segments were extracted from GENCODE (vM23). HTSeq was used to count reads that map to exonic or intronic segments for each gene.

### mTAIL-seq and data processing

mTAIL-seq library was constructed as previously described (Lim et al., 2016) with an additional gel purification after 5′ adapter ligation. We used STAR to align read1 to the reference genome (mm10) to get the detail alignment. Then, paired reads were mapped using STAR to allow soft-clipping to the alignments. We filter the read2 by removing read without the tag (GTCAG) on positions 15–25 nt from the 5′-end. Those consecutive poly(A) on filtered read2 not entirely aligned to the genome were considered as putative poly(A) tags. Using reference genome annotation (GENCODE vM23), we assigned these putative poly(A) tags by considering read1 alignment overlapped with annotated genes. Then we could calculate the poly(A) length for each poly(A) tag and assign these poly(A) length to each gene.

### Function enrichment analysis

For GO enrichment analysis, we used DAVID to estimate enrichment for each gene set (Huang et al., 2009). Enrichment Map was used to visualize the enrichment results (Merico et al., 2010). For GSEA analysis, we used the R package fgsea to calculate the enrichment score and *P*-value. The input gene sets (.gmt) were downloaded from g: profiler. The enrichment results were visualized using R.

## Supplementary material

Supplementary material is available at *Journal of Molecular Cell Biology* online.

## Supplementary Material

mjaa061_Supplementary_Data
