# Supplementary material for: RNF219 interacts with CCR4–NOT in regulating stem cell differentiation
Source: J Mol Cell Biol. 2020 Oct 26;12(11):894–905. doi: 10.1093/jmcb/mjaa061 (PMC7883825; doi:10.1093/jmcb/mjaa061)
Supplement: mjaa061_Supplementary_Data [file mjaa061_supplementary_data.pdf]

Fig S1

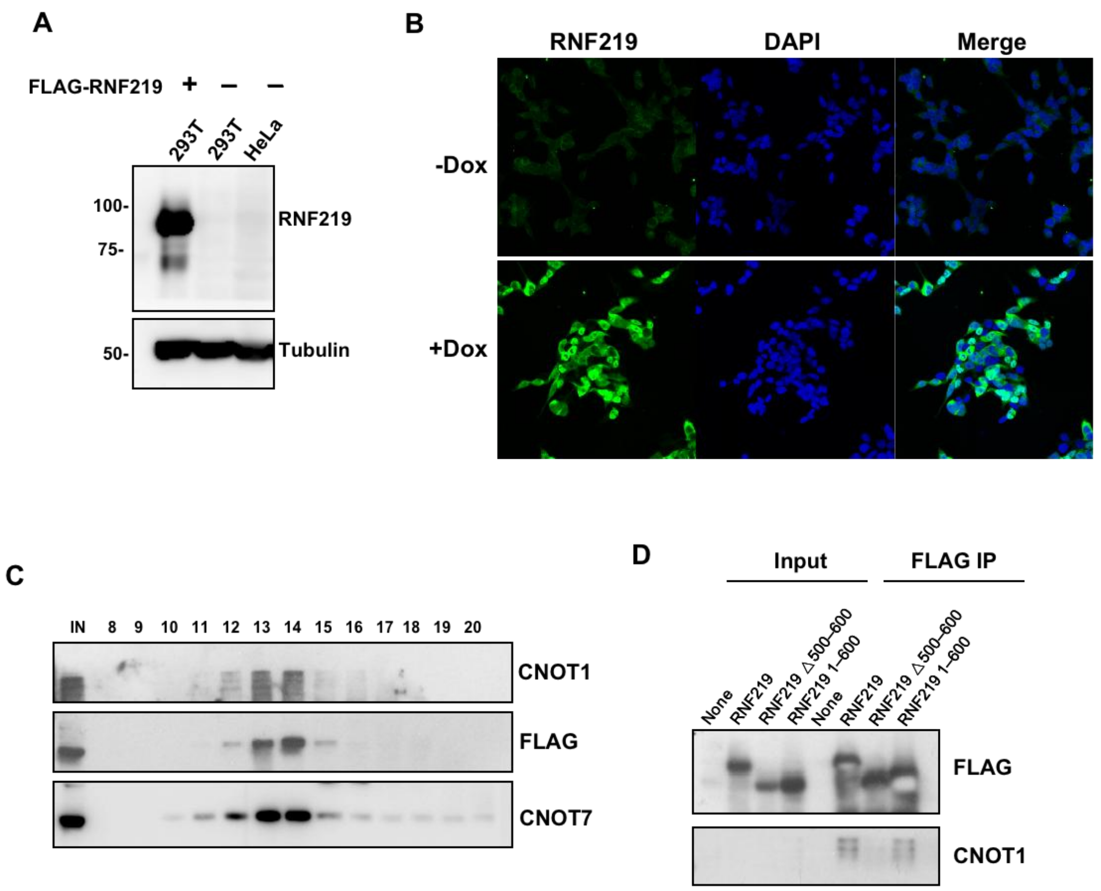

Supplementary Figure S1. Interaction of RNF219 with the components of CCR4-NOT.

(**A**) Western blot showing that the RNF219 antibody used in this study was able to recognize ectopically expressed FLAG-RNF219 in HEK-293 cells. (**B**) Immunofluorescence showing that the RNF219 antibody was able to recognize ectopically expressed FLAG-RNF219. (**C**) Size exclusion chromatography of nuclear extracts from the FLAG-RNF219 stable cell line demonstrating that majority of RNF219 co-eluted with the components of CCR4-NOT. (**D**) FLAG immunoprecipitations showing that deletion of aa 500–600 in RNF219 impaired RNF219 interaction with CNOT1.

Fig S2

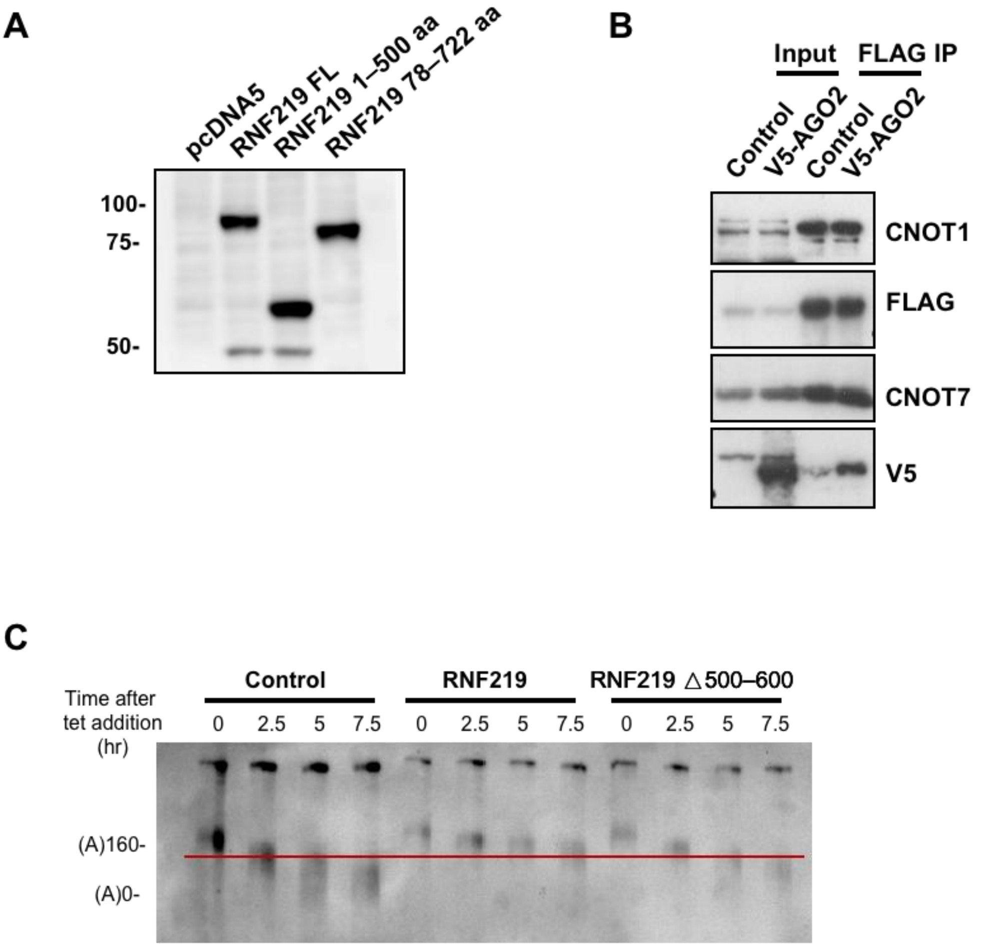

Supplementary Figure S2. Interaction of RNF219 with AGO2.

(**A**) Western blot showing overexpression of RNF219 full length, aa 1–500 and aa 78–722 truncation proteins used in deadenylation assays. (**B**) Interaction of RNF219 with AGO2. V5-AGO2 was expressed in FLAG-RNF219 stable cell lines. The cells were then subjected to lysis, and FLAG immunoprecipitation. (**C**) Deadenylation assay of the m^6A reporter following the overexpression of RNF219 full length and the aa 500–600 deletion mutant.

Fig S3

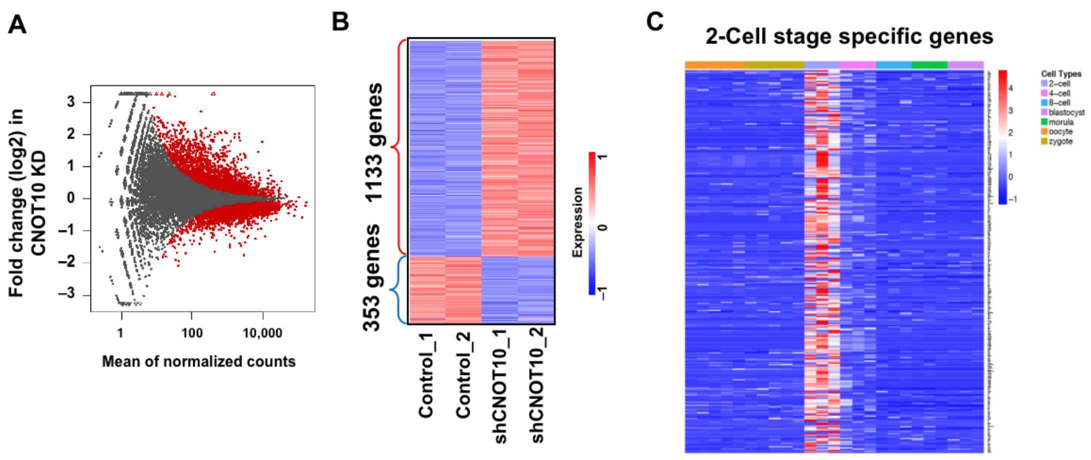

Supplementary Figure S3. Genome wide analyses of gene expression change after CNOT10 knockdown.

(**A**) MA plot showing differential expression of genes after knockdown of CNOT10 in mouse ES cells. The MA plot depicts the mean of normalized counts (x-axis) and log2 fold changes that calculated using DESeq2. The red dots represent genes with significance that the adjusted p-value < 0.05. (**B**) Heat maps showing expression levels of differential expressed genes (|Log2FC|>0.58) in CNOT10 knockdown mouse ES cells. (**C**) Heat maps showing the specificity ratio for each 2-cell stage genes in each cell type.

Fig S4

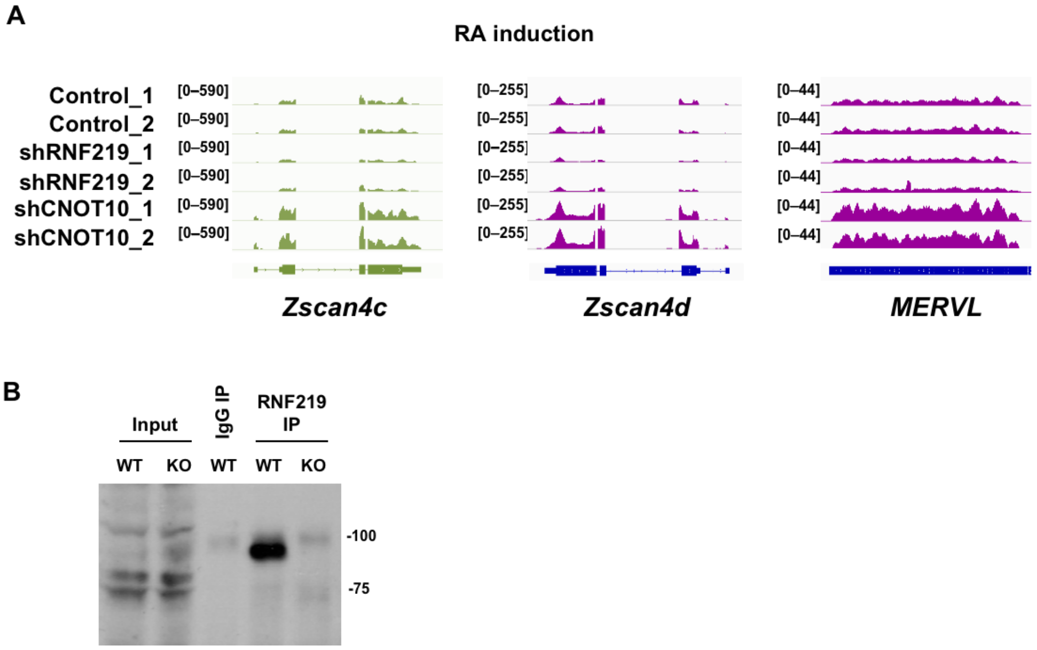

Supplementary Figure S4. Oppositely regulation of *Zscan4* and *MERVL* by RNF219 and CNOT10 in mouse ES cells treated with RA.

(**A**) Genome browse track file showing that the *Zscan4* family and *MERVL* were down-regulated after RNF219 knockdown, but up-regulated after CNOT10 knockdown in mouse ES cells treated with RA. (**B**) Validation of the RNF219 KO ES cell line generated in this study by immunoprecipitation, followed by western blot.
